# Long term mortality trends in people with severe mental illnesses and how COVID-19, ethnicity and other chronic mental health comorbidities contributed: a retrospective cohort study

**DOI:** 10.1017/S0033291724001843

**Published:** 2024-10

**Authors:** Jayati Das-Munshi, Ioannis Bakolis, Laia Bécares, Hannah K. Dasch, Jacqui Dyer, Matthew Hotopf, Rosie Hildersley, Josephine Ocloo, Robert Stewart, Ruth Stuart, Alex Dregan

**Affiliations:** 1 Department of Psychological Medicine, Institute of Psychiatry, Psychology & Neurosciences, King's College London, London, UK; 2 ESRC Centre for Society and Mental Health, King's College London, London, UK; 3 South London & Maudsley NHS Trust, London, UK; 4 Population Health Improvement UK (PHI-UK), UK; 5 Centre for Implementation Science, Health Service & Population Research Department, Institute of Psychiatry, Psychology & Neuroscience, King's College London, London, UK; 6 Department of Biostatistics and Health Informatics, Institute of Psychiatry, Psychology & Neuroscience, King's College London, London, UK; 7 Department of Global Health & Social Medicine, King's College London, London, UK; 8 NHS England & NHS Improvement (NHS-E/I), Black Thrive Global, UK; 9 National Institute for Health and Care Research (NIHR) Applied Research Collaboration (ARC) South London, Institute of Psychiatry, Psychology & Neuroscience, King's College London, De Crespigny Park, London, UK

**Keywords:** bipolar affective disorders, comorbidities, COVID-19, mortality, race/ethnicity, schizophrenia

## Abstract

**Background:**

People with schizophrenia-spectrum and bipolar disorders (severe mental illnesses; ‘SMI’) experience excess mortality. Our aim was to explore longer-term trends in mortality, including the COVID-19 pandemic period, with a focus on additional vulnerabilities (psychiatric comorbidities and race/ ethnicity) in SMI.

**Methods:**

Retrospective cohort study using electronic health records from secondary mental healthcare, covering a UK region of 1.3 million people. Mortality trends spanning fourteen years, including the COVID-19 pandemic, were assessed in adults with clinician-ascribed ICD-10 diagnoses for schizophrenia-spectrum and bipolar disorders.

**Results:**

The sample comprised 22 361 people with SMI with median follow-up of 10.6 years. Standardized mortality ratios were more than double the population average pre-pandemic, increasing further during the pandemic, particularly in those with SMI and psychiatric comorbidities. Mortality risk increased steadily among people with SMI and comorbid depression, dementia, substance use disorders and anxiety over 13-years, increasing further during the pandemic. COVID-19 mortality was elevated in people with SMI and comorbid depression (sub-Hazard Ratio: 1.48 [95% CI 1.03–2.13]), dementia (sHR:1.96, 1.26–3.04) and learning disabilities (sHR:2.30, 1.30–4.06), compared to people with only SMI. COVID-19 mortality risk was similar for minority ethnic groups and White British people with SMI. Elevated all-cause mortality was evident in Black Caribbean (adjusted Rate Ratio: 1.40, 1.11–1.77) and Black African people with SMI (aRR: 1.59, 1.07–2.37) during the pandemic relative to earlier years.

**Conclusions:**

Mortality has increased over time in people with SMI. The pandemic exacerbated pre-existing trends. Actionable solutions are needed which address wider social determinants and address disease silos.

## Background

The COVID-19 pandemic worsened pre-existing inequalities in health outcomes and healthcare access for the most disadvantaged groups, particularly in countries with the highest levels of social inequities (Bambra, Riordan, Ford, & Matthews, 2020). Racially minoritized communities, and people living with mental disorders, including severe mental illnesses (SMI), such as schizophrenia-spectrum and bipolar affective disorders, were noted to be at a higher risk of SARS-Cov2 infection (Mathur et al., 2021; Yang et al., 2020), as well as poorer outcomes (Mathur et al., 2021; Vai et al., 2021; Yang et al., 2020) and death (Das-Munshi et al., 2021a; Fond et al., 2021; Mathur et al., 2021; Vai et al., 2021) following infection. Chronic comorbid physical health conditions such as cardiovascular disease and diabetes have been shown to exacerbate mortality following SARS-CoV-2 infection (Chudasama et al., 2020). A growing body of work has also highlighted the additional contributions which long-term mental health conditions play in heightening the risk of death following infection (Carmona-Pírez et al., 2022; Chudasama et al., 2020), such as substance use disorders (Vai et al., 2021; Wang, Kaelber, Xu, & Volkow, 2021), dementia (Atkins et al., 2020; Clift et al., 2020; Hariyanto, Putri, Arisa, Situmeang, & Kurniawan, 2021), anxiety (Carmona-Pírez et al., 2022), depression (Atkins et al., 2020; Chudasama et al., 2020; Clift et al., 2020) and learning disabilities (Vai et al., 2021). The interplay of these comorbidities with other vulnerability factors, associated with a higher risk of death following SARS-CoV-2 infection, in people already living with SMI, remains unclear.

Prior to the pandemic there were already concerns that mortality risks in people with SMI had increased over time (Saha, Chant, & McGrath, 2007), with investigators reporting an increase in relative rates of death in people with SMI compared to population controls, further suggesting growing inequalities over time (Hayes, Marston, Walters, King, & Osborn, 2017). The impact of the COVID-19 pandemic on potentially exacerbating underlying longer-term trends, in addition to exacerbating other inequalities, for example with respect to ethnicity, remains unclear (Impara et al., 2022), despite a concern that across high income international contexts, Black and other racially minoritized people have been at a higher risk of COVID-19 infection and adverse outcomes, including death (Pan et al., 2020).

Using data from a well-defined ethnically diverse clinical population with SMI, followed for 14 years, including one year during the pandemic, we sought to assess the following research questions:

1. How have mortality trends changed in people with SMI over time?
2. In people with SMI, how has the presence of comorbid mental health conditions or minority ethnic group status further impacted inequities?
3. How did the COVID-19 pandemic impact pre-existing mortality trends in people with SMI?
4. During the pandemic, what role did co-existing mental health comorbidities and ethnicity play in people with SMI, with respect to all-cause and cause-specific (COVID-19 and non-COVID19) mortality?

## Methods

### Participants, setting, and study design

We used electronic health records data from one of Europe's largest secondary mental health providers, South London & Maudsley Trust (SLaM Trust) (Perera et al., 2016). SLaM Trust provides near-complete secondary mental healthcare to a catchment of 1.3 million people residing in southeast London. The Clinical Records Interactive Search (CRIS) system was established in 2007 and is an ethically approved interface which enables researchers to access de-identified patient care records for approved research projects. CRIS contains detailed clinical, therapeutic and sociodemographic routinely collected data from a real-world setting.

Mental health diagnoses are assigned to people under the care of SLaM Trust by clinicians, according to the International Classification of Mental Disorders-10 (ICD-10) (World Health Organization, 2011). Using these codes in structured fields, supplemented with validated Natural Language Processing (NLP) algorithms to further text-mine patient case records (Das-Munshi et al., 2021a; Perera et al., 2016), we created a cohort of individuals with F20–F29 codes (schizophrenia, schizotypal and delusional disorders, herewith referred to as schizophrenia-spectrum disorders) and F30.0 (manic episode) and F31.0 (bipolar affective disorders) codes. Individuals with pre-existing dementia diagnoses were excluded.

We conducted a retrospective cohort study following individuals from their date of SMI diagnosis, or study start date (1 Jan 2007) if diagnosed before 2007, to 1st October 2021 (or death [‘all-cause mortality’]), whichever came first. We next assessed cause-specific mortality (deaths from COVID-19 and deaths from all other/ non-COVID-19 causes) in individuals followed from the start of the pandemic (30th January 2020, when the World Health Organization declared a Public Health Emergency of International Concern [PHEIC]) (World Health Organization, 2020) until the end of the study on 17th February 2021 (or death, whichever came first). As COVID-19 did not exist prior to the pandemic, cause-specific mortality models to assess the risk of death from COVID-19, could not be undertaken using pre-pandemic data.

Participants were included in the study if they were over the age of 18 at the time of diagnosis and had a relevant clinical SMI diagnosis, with complete data on all relevant indicators (see STROBE flow diagram, online Supplementary Fig. S1).

### Measures

#### Demographic and social measures

Variables taken from structured fields included sex (male/ female) and marital status (married, cohabiting, divorced, separated, single). In addition, ethnicity data, subsequently categorized according to Office for National Statistics (ONS) criteria, were used. This resulted in the following groups: White British, Irish, Black Caribbean, Black African, Indian, Pakistani, and Bangladeshi. Due to low numbers, the Indian (*n* = 544), Pakistani (*n* = 265) and Bangladeshi (*n* = 150) groups were aggregated into a ‘South Asian’ group. Due to concerns around within-group heterogeneity, ‘Other Asian’ (*n* = 1053), ‘Other ethnicity’ (*n* = 1659), ‘Other White’ (*n* = 2180) and ‘Other mixed ethnicity’ (*n* = 245) groups were removed. Area deprivation, classified according to the Index of Multiple Deprivation (IMD) (Noble, Wright, Smith, & Dibben, 2006) was estimated for each participant by linking participant lower super output area (LSOA) codes to the relevant deprivation indicator. LSOAs are small administrative geographical areas in the UK which have a mean population of 1500 people (Department for Communities and Local Government, 2015). When linked to the IMD indicator, a measure for area-level deprivation (capturing deprivation across the domains of income, employment, education, health, crime, housing, and living environment) is derived, providing a relative measure of a local area's deprivation levels across these domains, compared with other areas nationally (Department for Communities and Local Government, 2015). This variable was subsequently categorized into quintiles (ranging from most deprived [1st quintile] to least deprived [5th quintile]) for analyses. We used date of birth to calculate age of participants, which was handled as a time-varying covariate in analyses (see below).

#### SMI and co-existing morbidities

Date of first SMI diagnosis was used to calculate years living with severe mental illnesses, treated as a time-varying covariate (detailed below). Additional comorbidities with SMI were identified using clinical ICD-10 diagnoses entered into structured fields, further supplemented by validated NLP algorithms applied to the free text recorded in the clinical notes (Das-Munshi et al., 2021a; Perera et al., 2016). Comorbid mental health diagnoses used in the analyses included comorbid depression (occurring/ recorded at any time (ICD-10 codes: F3*), comorbid/ post-SMI dementia (recorded after but not before the index SMI diagnosis) (ICD-10 codes: F00–F03), comorbid substance and alcohol use disorders, occurring/ recorded at any time (ICD-10 codes: F10–F19), comorbid learning disabilities, occurring/ recorded at any time (ICD-10 codes: F70–F79), and comorbid neurotic, stress-related and somatoform disorders/ anxiety disorders occurring/ recorded at any time (ICD-10: F40–F48).

#### Outcomes

Using a data linkage of SLaM patient-level health records to national deaths records information enabled through National Health Service (NHS) numbers, which are unique patient identifiers, we were able to assess all-cause mortality and date of death for all cohort members within the study, from 1st January 2007 to 1st October 2021.

For cause-specific mortality, we used data from a linkage to ONS death certificate records to identify deaths where COVID-19 had been mentioned anywhere on the death certificate (ICD-10 codes U07.1 or U07.2), contrasted against deaths from ‘all other causes’. Mortality from COVID-19 was identified to a slightly earlier date of 17th February 2021.

### Statistical methods

To address research questions 1 and 2, we assessed deaths in the study population, using a cohort study design. We used a parametric regression survival model with an exponential survivor function, equivalent to Poisson regression (Kirkwood & Sterne, 2003), to assess all-cause mortality, taking into account the time-changing covariates of age and time period (years). Through Lexis expansion (Clayton & Hills, 1993), age was split into three groups (15–45/45–65/65+). A quadratic term for age was found to have a better fit to the data, using Likelihood Ratio Tests (LRTs) therefore age in all models was subsequently added as a quadratic term to regression models. Using Lexis expansion, we also created a ‘time since diagnosis’ variable, which was categorized into four time periods from the date of SMI diagnosis (<1, 1–5, 5–10, 10+ years from diagnosis). To capture period effects, we created a third time-varying covariate (‘years’) by Lexis expansion, to create consecutive time periods, each spanning 24 months, from 1 Jan to 31 Dec of the following year (2008–2009, 2010–2011, 2012–2013, 2014–2015, 2016–2017, 2018–2019). For the period of the COVID-19 pandemic (2020–2021), dates spanned from 1st January 2020 to 1st October 2021 (22 months). For the first period in this variable (−2007), dates spanned 12 months from 1 Jan–31 Dec 2007, when records first started. For analyses assessing time trends in all-cause mortality (described below), the midpoint of the study (2014–2015) was taken as the reference period. Other *a priori* confounders in all models were: sex, marital status, and area deprivation, alongside age and time since diagnosis.

We assessed an interaction term between ethnicity and time period (year). We then calculated the adjusted risk of death for each ethnic minority group per time period, taking the study midpoint (2014–2015) for the minority ethnic group in question, as the reference period. To assess whether mental health comorbidities (depression, dementia, learning disabilities, alcohol and substance use disorders, and anxiety disorders) modified the risk of all-cause mortality in people with SMI during the pandemic, we fitted an interaction term between each comorbidity with time period, taking the study midpoint (2014–2015) as the reference period. Models were assessed using Likelihood Ratio Tests (LRTs).

To address research questions 3 and 4, we calculated age and sex-standardized mortality ratios (SMRs) using deaths in the sample, over 2019 (pre-pandemic period) and 2020 (spanning the pandemic, from 1 Jan to 31 Dec), respectively. We calculated age in ten-year bands for the sample, anchored to the midpoints of 2019 and 2020 respectively, and stratified by sex (male/female). Deaths and age and sex structure of the study population were used to calculate ‘observed’ totals. ONS deaths and mid-year population estimates for England and Wales for 2019 and 2020 were used as a reference population, to derive ‘expected’ totals. The indirect method of standardization was used to derive SMRs with 95% confidence intervals, using the *istdize* package in STATA.

To address research question 4, we followed the cohort from the start of the pandemic on 30^th^ January 2020 until death or the end of the study on 1st February 2021 (whichever came first), and assessed cause-specific mortality outcomes using a competing risks models, with a modified Cox proportional hazards regression model (Fine & Gray, 1999). In these models, the sub-Hazard Ratio (sHR) for death from COVID-19 was assessed while taking into account the competing risk of death from all other/non-COVID-19 causes. We then repeated the analyses, specifying the risk of death from all other/non-COVID-19 causes, taking into account the competing risk of death from COVID-19. Exposures assessed in these models were mental health comorbidities and ethnicity. All cause-specific mortality analysis models adjusted for *a priori* confounders (age, sex, ethnicity, marital status, area deprivation, and time since diagnosis). Schoenfeld-like residuals were examined for each regressor in competing risks regression models, to verify proportional hazards assumptions. Analyses were performed in Stata MP version 15.1 (StataCorp, 2017), with the *stsplit* command used for Lexis expansion, the *streg* command to undertake Poisson regression for all-cause mortality, and the *stcrreg* command to implement competing risks regression for cause-specific mortality analyses.

For missing data we undertook sensitivity analysis using multiple imputations with chained equations, replacing missing values with imputed values, under Missing at Random assumptions. Ten imputation cycles were undertaken, using the *mi impute* command in STATA, with derived datasets stratified by SMI comorbidities. The imputation regression contained the outcome (deaths), all regression covariates (ethnicity, sex, marital status, area deprivation) and additional auxiliary variables (other ICD-10 diagnoses, including dementia, substance use disorders, personality disorders, intellectual disorders, F3*, F4*, F50, and F8* diagnoses). Estimates were then combined across imputed datasets, using Rubin's rules.

## Results

The cohort comprised 22 361 people with SMI, followed from 2007 to 2021, with a median follow up time of 10.6 years. Table 1 displays characteristics of the study sample. Just under half (47%) of the sample were of a minority ethnic background, and a significant proportion (85%) were single, widowed or in disrupted relationships. More than half (55%) of the sample had comorbid depression and just under a quarter had comorbid alcohol and substance use disorders (22%) or comorbid anxiety (24%). Online Supplementary Fig. S1 shows the flow of participants.

**Table 1.** Demographic characteristics of the sample

	All-cause mortality sample (2007–2021)				Cause specific mortality sample (2020–2021)					
	Total sample		Deaths (all causes)		Total sample		Deaths (non-COVID19)		Deaths (COVID-19)	
	*N* (22 361)	(%)	*N* (4104)	(%)	*N* (18 514)	(%)	*N* (549)	(%)	*N* (127)	(%)
Ethnicity										
White British	11 883	53	2574	63	9474	51	319	58	–	–
Irish	648	3	215	5	442	2	16	3	–	–
Black Caribbean	5689	25	862	21	4890	26	132	24	–	–
Black African	3182	14	304	7	2896	16	63	11	–	–
South Asian	959	4	149	4	812	4	19	3	–	–
Relationship status										
Married/ cohabiting	3346	15	682	17	2693	15	79	14	23	18
Divorced/ separated/ single/ widowed	19 015	85	3422	83	15 821	85	470	86	104	82
Sex										
Male	11 676	52	2121	52	9737	53	298	54	57	45
Female	10 685	48	1983	48	8777	47	251	46	70	55
Area deprivation (IMD quintiles)										
1 (Most deprived)	5222	23	1022	25	4262	23	138	25	27	21
2	5068	23	870	21	4263	23	121	22	29	23
3	4722	21	883	22	3907	21	125	23	23	18
4	4340	19	762	19	3627	20	99	18	31	24
5 (Least deprived)	3009	13	567	14	2455	13	66	12	17	13
SMI Comorbidities										
Depression	12 313	55	1928	47	10 475	57	281	51	79	62
Dementia	1238	6	727	18	647	3	113	21	28	22
Learning disabilities	1190	5	172	4	1039	6	22	4	14	11
Substance and alcohol use disorders	4919	22	679	17	4259	23	119	22	14	11
Anxiety disorders	5258	24	550	13	4679	25	107	19	21	17

– suppressed due to low numbers in cells.

Table 2 displays age and sex-standardized mortality ratios (SMRs) for the sample in 2019 and then in 2020. SMRs were elevated more than two-fold during 2019 (prior to the pandemic) and remained at this level during 2020, reflecting that excess mortality in the SMI sample remained more than double the population average, even during a year of unprecedented excess mortality in the general population, when deaths in the general population reference would have substantially risen also. People with SMI and comorbid depression or dementia experienced significantly elevated SMRs during the pandemic. People with SMI and learning disabilities experienced substantially elevated SMRs during the pandemic (SMR: 6.08, 95% CI 4.13–8.63). Mortality risk was elevated more than two-fold in people with SMI, across all minority ethnic groups, and in the White British group, during the pandemic.

**Table 2.** Age and sex-standardized mortality ratios (SMRs) (all-cause mortality) pre-pandemic (2019) and during the COVID-19 pandemic (2020)

		Year	Observed	Expected	SMR	(95% CI)	
Full sample	SMI	2019	339	138	2.46	(2.21	2.74)
		2020	415	160	2.59	(2.35	2.86)
SMI Comorbidities	Depression	2019	176	66	2.66	(2.28	3.08)
		2020	296	79	3.77	(3.35	4.23)
	Dementia	2019	52	33	1.58	(1.18	2.07)
		2020	119	36	3.26	(2.70	3.90)
	Learning disability	2019	15	4	3.36	(1.88	5.53)
		2020	31	5	6.08	(4.13	8.63)
	SUDs	2019	73	13	5.67	(4.45	7.13)
		2020	108	16	6.88	(5.65	8.31)
	Anxiety	2019	66	23	2.91	(2.25	3.70)
		2020	97	26	3.70	(3.00	4.51)
Ethnicity	White British	2019	211	75	2.82	(2.46	3.23)
		2020	245	87	2.83	(2.49	3.21)
	Irish	2019	25	6	3.86	(2.50	5.70)
		2020	16	6	2.54	(1.45	4.12)
	Black Caribbean	2019	73	37	1.98	(1.56	2.50)
		2020	99	43	2.31	(1.88	2.81)
	Black African	2019	19	14	1.36	(0.82	2.12)
		2020	41	17	2.36	(1.69	3.20)
	South Asian	2019	11	6	1.89	(0.94	3.38)
		2020	14	7	2.06	(1.13	3.46)

SUDs, Substance and alcohol use disorders.

**Figure 1.**
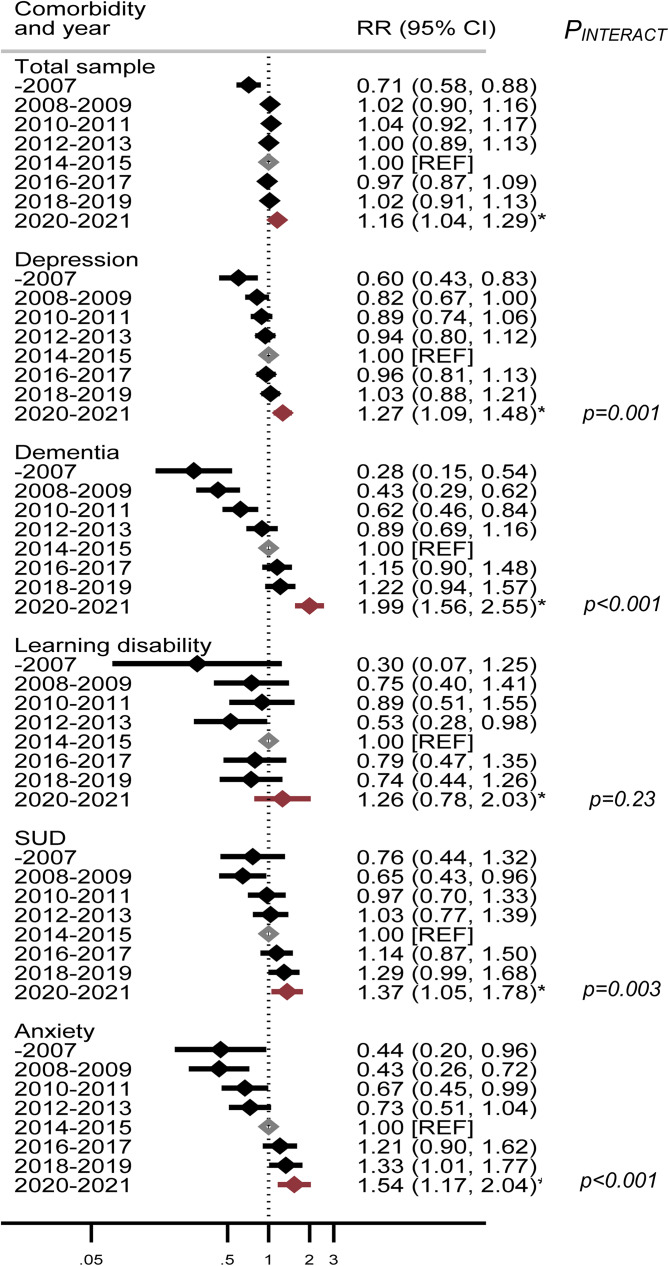
All-cause mortality in people with severe mental illness (SMI). Cohort followed from 2007 to 2021, stratified by comorbidities and year. **Legend:** *N* = 22 361; Displayed adjusted rate ratios (RRs) are for each comorbid condition with SMI, relative to the 2014–2015 reference period. *Indicates adjusted rate ratios for all-cause mortality from 30th January 2020–2021 (first year of the COVID-19 pandemic). All estimates have been adjusted for age, marital status, gender, area deprivation, ethnicity and time since SMI diagnosis. SUDs, Substance and alcohol use disorders. *P*_INTERACT_ denotes comorbidity by period interactions, which were assessed with Likelihood Ratio Tests (LRTs). Association of time period with mortality risk in the total sample was *p* = 0.002 (LRT).

Figure 1 displays the association of time periods (from 2007–2021) with all-cause mortality risk for the SMI sample as a whole, and stratified by comorbidity, compared to the midpoint (2014–2015) reference period. There was strong evidence supporting comorbidity by time interactions across all comorbid conditions except for learning disabilities. Earlier time periods were associated with lower mortality compared to the reference year, and later periods, including the pandemic years (2020–2021) were associated with higher mortality rates. The risk of death during the pandemic was substantially elevated in people with SMI and comorbid dementia (aHR: 1.99 [95% CI 1.56–2.55]), albeit consistent with a prior pre-existing trend toward increasing mortality risk over time, since 2007. Likelihood ratio tests were used to assess the fit of models with time period as a categorical variable (i.e. no assumption about how log rate changed with time) and time period as a quantitative variable (i.e. linear assumption of log rate change). Each of the LRTs provided stronger support for ‘time period’ as a categorical variable over a linear variable (online Supplementary Table S1).

**Figure 2.**
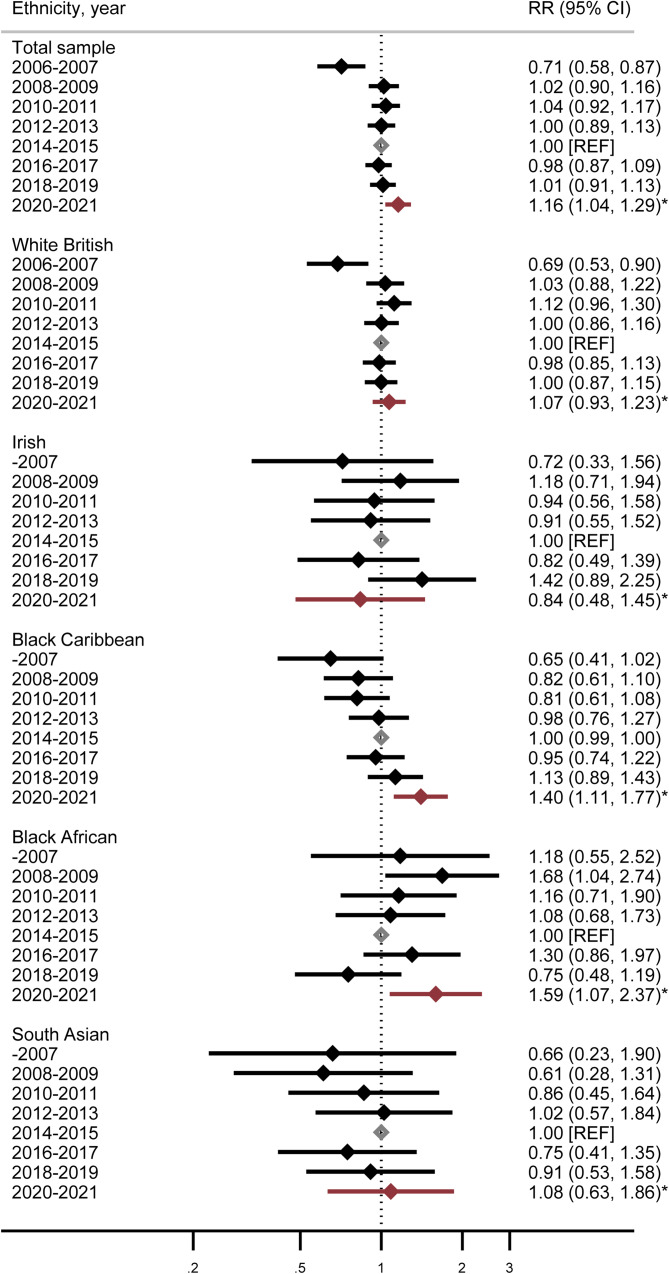
All-cause mortality in people with severe mental illness (SMI). Cohort followed from 2005 to 2021; full sample and stratified by ethnicity and year. **Legend:** *N* = 22 361; Displayed adjusted rate ratios (RRs) are for the total sample and stratified by ethnicity relative to the 2014–2015 reference period. *Indicates adjusted rate ratios for all-cause mortality from 30th January 2020–2021 (first year of the COVID-19 pandemic). All estimates have been adjusted for age, marital status, gender, area deprivation and time since SMI diagnosis. Ethnicity by period interactions were *p* = 0.0065 (Likelihood ratio tests, LRTs). Association of time period with mortality risk in the total sample was *p* = 0.002 (LRT).

Figure 2 displays the association of SMI with all-cause mortality, across the total sample, and stratified by ethnicity, from 2007 to 2021. Displayed estimates are again relative to 2014–2015 (midpoint of the study sample), with estimates from the pandemic years (2020 onwards) displayed in red. There was strong evidence from Likelihood ratio tests (*p* = 0.0065) to suggest an ethnicity × period interaction (*v*. no interaction) for the models displayed in Fig. 2. Across the White British and Irish groups, all-cause mortality during the period spanning the pandemic (2020–2021) was overall similar to the reference period (2014–2015) and across preceding years. Compared with the reference period (2014–2015), there were moderate increases in all-cause mortality in the Black Caribbean group with SMI (aHR 1.40 [95% CI 1.11–1.76]) during the pandemic. All-cause mortality also increased in the Black African (aHR: 1.59 (95% CI 1.07–2.33]) group with SMI during the pandemic, relative to the pre-pandemic reference period.

Table 3 displays associations of comorbidity and ethnicity with all-cause and cause-specific mortality, for cohort members followed from the start of the pandemic on 30th January 2020. The presence of comorbid depression, dementia and learning disabilities were associated with an increased risk of death from COVID-19; this was substantial for comorbid dementia (sHR: 1.96 [95% CI 1.26–3.04; *p* < 0.001]) and learning disabilities (sHR: 2.30 [95% CI 1.30–4.06; *p* < 0.001]). Comorbid dementia was associated with an elevated risk of all-cause mortality as well as a higher risk of deaths from non-COVID-19 related causes. Comorbid substance/ alcohol use disorders were associated with a 31% increased risk of death from non-COVID-19 related causes, but not associated with an increased risk of COVID-19 mortality. There was no evidence of an association between ethnicity and all-cause and cause-specific mortality.

**Table 3.** Associations with cause-specific mortality (Cohort members followed from 30th January 2020 to 17th February 2021)

			All-cause mortality				Non-COVID19 mortality				COVID-19 mortality			
	*N* (18,514)	%	RR	(95 %)		*p* value	sHR	(95 %)		*p* value	sHR	(95 %)		*p* value
SMI comorbidity														
Depression	10,475	57%	1.01	(0.86	1.18)	0.91	0.93	(0.78	1.11)	0.40	1.48	(1.03	2.13)	0.03
Dementia	647	3%	2.46	(2.01	3.01)	*p* < 0.001	2.62	(2.10	3.26)	*p* < 0.001	1.96	(1.26	3.04)	*p* < 0.001
Learning disability	1,039	6%	1.01	(0.72	1.42)	0.94	0.75	(0.49	1.15)	0.19	2.30	(1.30	4.06)	*p* < 0.001
SUD	4,259	23%	1.19	(0.97	1.45)	0.09	1.31	(1.06	1.61)	0.01	0.70	(0.39	1.26)	0.24
Anxiety disorders	4,679	25%	0.95	(0.78	1.15)	0.58	0.97	(0.78	1.20)	0.78	0.86	(0.54	1.38)	0.54
Ethnicity														
White British	9,474	51%	1.00	REF			1.00	REF			1.00	REF		
Irish	442	2%	0.81	(0.52	1.27)	0.66	0.80	(0.49	1.33)	0.56	0.87	(0.32	2.38)	0.34
Black Caribbean	4,890	26%	1.00	(0.83	1.21)		0.96	(0.78	1.18)		1.23	(0.80	1.88)	
Black African	2,896	16%	0.85	(0.66	1.10)		0.86	(0.65	1.13)		0.81	(0.42	1.57)	
South Asian	812	4%	0.93	(0.64	1.37)		0.75	(0.47	1.18)		1.77	(0.92	3.42)	

**Key:** RR, Rate ratios; sHR, sub-Hazard Ratios; *p* values derived from Wald tests. SUD, Substance and alcohol use disorders; SMI, Severe Mental Illness. Estimates are adjusted for ethnicity, marital status, sex, area deprivation, years diagnosed with SMI and age.

### Sensitivity analysis

To assess if all-cause mortality trends (Figs 1 and 2) were biased by people with more severe mental health conditions coming into contact with mental health services in later years compared to earlier years, we conducted *post hoc* sensitivity analyses. Analyses were repeated, restricting the sample to people with SMI diagnoses prior to 31st December 2010, only. Online Supplementary Figs S2 and S3 display these analyses and confirm that observed mortality trends remained robust to these changes in the sample inclusion criteria.

Sensitivity analyses with multiple imputations for missing values under Missing At Random assumptions were also undertaken for all-cause mortality estimates (online Supplementary Table S2). Estimates for the association between SMI comorbidities and all-cause mortality were similar to those derived through complete case analyses (in Fig. 2), although imputed estimates were marginally higher for all-cause mortality risk during the pandemic (2020–21). For example, during 2020–2021, the association of depression comorbid with SMI with all-cause mortality was IRR: 1.27 (95% CI 1.09–1.48) in complete case models and IRR:1.29 (95% CI 1.12–1.48) in imputed models (online Supplementary Table S2).

Finally, to assess if changes to the SMI definition impacted associations, we re-ran analyses assessing associations with all-cause mortality, restricting to schizophrenia-spectrum (ICD-10 F2*) diagnoses only (see online supplementary material Figs S4 and S5). This restriction had a minimal effect on associations, although some SMI comorbidities showed a marginally larger association with all-cause mortality with this restricted definition; for example the association of depression comorbid with a restricted definition of SMI (ICD-10 F2*) with all-cause mortality was IRR 1.39 (95% CI 1.14–1.70) in these models (compared to: IRR 1.27 (95% CI 1.09–1.48), for the ‘broader’ SMI definition models).

## Discussion

In a cohort of 22 361 people with schizophrenia-spectrum and bipolar disorders, from a large geographical urban catchment area, we found that people living with SMI and comorbid depression, dementia, substance use disorders and anxiety have experienced worsening mortality outcomes over time, with the pandemic in 2020 further exacerbating adverse trends. From 2020, all-cause mortality in Black Caribbean and Black African people with SMI was also elevated, compared to pre-pandemic years. We found that deaths from COVID-19 were especially elevated in people with SMI and comorbid dementia, learning disabilities and depression, whilst deaths from all other/ non-COVID-19 causes during the pandemic were elevated in people with SMI and comorbid dementia or comorbid substance and alcohol use disorders.

In our analyses which focused on the period of the pandemic (2020–2021), we did not find that people of racially minoritized communities with SMI had an elevated risk of all-cause or cause-specific mortality, relative to White British people with SMI. This latter finding should be understood within the wider context, where we found that standardized mortality ratios were more than double the population average pre-pandemic, and increased further during the pandemic, particularly in those with SMI and mental health comorbidities. The increased risk of death relative to the general population was observed across all minority ethnic groups with SMI, alongside the White British group with SMI. Our findings are consistent with a recent report from a whole population analysis, which highlighted high mortality in people living with schizophrenia-spectrum disorders (*v.* those without), especially when comorbid with neurotic/ stress-related disorders (including anxiety disorders) and substance use disorders (Plana-Ripoll et al., 2020). Our findings extend this and other previous analyses (Das-Munshi et al., 2021a; Fond et al., 2021; Vai et al., 2021), by confirming that the risk of all-cause mortality further increased in the SMI sample during the pandemic, compared to pre-pandemic years. Our findings are consistent with previous studies which have highlighted a worsening mortality gap in people with SMI over time (Hayes et al., 2017; Saha et al., 2007). Our findings are consistent with previous work, which has highlighted that the presence of severe mental disorders has a significant impact on mortality risk, and this inequity is similarly experienced in minority ethnic and in White British groups (Das-Munshi et al., 2021b). The observed inequities in mortality outcomes observed in people with severe mental disorders may highlight the extreme levels of social exclusion experienced by this group, potentially exacerbated by higher levels of poverty. These findings also suggest a potential ‘cross-cutting’ adverse effect of severe mental illnesses on physical health outcomes, and is consistent with a previous study where we observed life expectancy in people with SMI to be worse than that of the general population residing in the most deprived areas of the UK (Das-Munshi et al., 2021b). In previous work, a range of factors have been implicated as potentially playing a role in premature mortality in SMI. This has included concerns around the quality of care which people with SMI receive, which may be poorly integrated or ‘siloed’ between physical health and mental health services, the impact of stigma, discrimination and diagnostic overshadowing, and the role of SMI itself, in potentially exacerbating the ability to attend to self-care and manage physical health challenges (O'Connor et al., 2023).

The findings relating to excess mortality from COVID-19 in people with SMI and other comorbidities may reflect the challenges that people with multiple long-term conditions experienced in attempting to access different health services, also apparent in minority ethnic groups (Bécares & Das-Munshi, 2013), and further exacerbated during the pandemic, particularly where care delivery may have been siloed (Ashworth, Schofield, & Das-Munshi, 2017; Topriceanu et al., 2021). The findings relating to people with SMI and comorbid learning disabilities or dementia, potentially reflects concerns raised during the early days of the pandemic, noted across multiple international contexts, that COVID-19 outbreaks and deaths particularly impacted people living in group residential care homes (Alacevich et al., 2021; Burton et al., 2020; Webster, 2021), which is more likely in people with these long-term conditions. In the UK, concerns were raised that people living in residential care homes were being put at an additional risk of contracting and dying from COVID-19, due to a lack of access to testing, personal protective equipment (PPE) for staff, and the discharge of people with COVID-19 from hospitals to residential group care homes, further seeding COVID-19 outbreaks amongst highly vulnerable populations, during the early months of the pandemic (Public Health England, 2021). Similar concerns were implicated in older people admitted to in-patient psychiatric units (Livingston et al., 2020). Specific health-related behaviors (smoking, physical inactivity) and treatment-related factors, which were already a concern prior to the pandemic, may have also been important drivers of the growing mortality gap between people with SMI and the general population (Dregan et al., 2020). COVID-19 lockdown restrictions may have further aggravated the prevalence of these factors within SMI populations.

In the UK, the period of the study spanned several lockdown periods. The lockdowns were associated with the sudden closure of community centers, the removal of face-to-face appointments, and prolonged periods of isolation at home with restrictions placed on social gatherings. These are now well documented as having adversely impacted upon people's social connections, support, networks and were linked to increased isolation and loneliness (Moreno-Agostino et al., 2022; Varga et al., 2021), and shown to have been further exacerbated in SMI populations (Heron et al., 2022). Loneliness and social isolation have been implicated in playing a role in increases in levels of alcohol and substance use in populations, over the period of the pandemic (Kim et al., 2020). In general population studies (Daly & Robinson, 2021) and in populations with known pre-existing alcohol use disorders (Kim et al., 2020), levels of alcohol use potentially increased during the pandemic. Our findings indicate that in people living with SMI and co-occurring alcohol or substance use disorders, deaths from all other/non-COVID-19 were elevated during the pandemic. It remains unclear if this finding relates to an increase in hazardous or harmful alcohol/substance use in people living with SMI, and/ or if this finding was a result of access to healthcare being reduced (either due to delayed or reduced health seeking, or because of service suspensions/ closures and transition to remote/telecare models) (Bakolis et al., 2021).

Strengths of our study include the retrospective cohort design which enabled follow up with linkage to all-cause and cause-specific mortality in people living with SMI, tracking deaths for over a decade prior to the start of the pandemic, and then following people with SMI for a full year after the pandemic was first declared. The linkage to death certificate information ensured accurate tracing with high levels of completeness for cause of death and for ascertainment of deaths due to COVID-19 in the sample. The use of electronic health records from a large ethnically diverse catchment area also ensured large sample sizes; local mandatory recording of ethnicity ensured high levels of completeness of this variable, whereas high levels of incomplete/ missing data on this variable have been a concern in previous research. Furthermore, the accuracy of the clinical mental health diagnoses in these records was ensured by the use of text mining of the clinical notes, using validated algorithms (Das-Munshi et al., 2021a). Our findings also remained robust to a range of sensitivity analyses, designed to assess the impact of potential bias on observed associations.

Deaths from COVID-19 are known to be elevated in people living with underlying physical health conditions, however we did not have access to this data at the time of this study. Cardiovascular disease, metabolic syndrome, respiratory and other physical health conditions, may mediate the association between SMI and mortality (Das-Munshi et al., 2017; Firth et al., 2019; Gronholm et al., 2021), and would need consideration through appropriate analyses (Lin, Young, Logan, & VanderWeele, 2017). This could be explored in the future and may shed further light on potential mechanisms. We were able to adjust for area deprivation in these analyses, however access to data on individual-level deprivation and poverty indices, which have been shown to have been important in deaths from COVID-19 (Bambra et al., 2020) were not available in the present study, and is a general limitation impacting most studies using electronic health records. We will be exploring accessing these additional data through methodological innovations such as data linkage (Cybulski et al., 2023) and text mining (Chilman et al., 2021) in the future. Our study spanned the entire population of southeast London (population of approximately 1.3 million residents) served by the mental health Trust. In the UK access to health care is free at point of contact through the National Health Service (NHS), although it is possible that some people living with SMI in the catchment of the study may have accessed care outside of the NHS privately; however, in reality this is likely to be a small number and should not have biased findings. Our study has good generalizability to other urban and metropolitan regions in the UK but is less generalizable to rural areas.

Increasingly, the notion of *syndemics,* first used to describe the socially patterned clustering of HIV and substance use disorders (Singer, 2000), has been extended to considering the impact of COVID-19 on marginalized and excluded populations (Bambra et al., 2020). The impacts of the pandemic on vulnerable groups has been implicated as heavily socially determined, with COVID-19 infection further magnifying the impact of pre-existent conditions, leading to excess mortality and adverse health outcomes (Bambra et al., 2020) in groups who were already marginalized, including minority ethnic groups. Co-occurring chronic disease are social patterned (Singer, Bulled, Ostrach, & Mendenhall, 2017), concentrated in the most disadvantaged (Bambra et al., 2020), with mutually enhancing adverse health effects (Bambra et al., 2020; Singer et al., 2017). Within this framework, it may be possible to understand the impact of co-occurring health conditions (such as dementia and learning disabilities) in people with SMI, which may have further exacerbated inequities both before, and during, the pandemic (Fond et al., 2021; Nemani et al., 2021). Future work should explore these intersections in SMI populations and may be enhanced through qualitative exploration of people's experiences during the pandemic (Impara et al., 2022).

In conclusion, our findings confirm that people living with SMI have experienced worsening mortality trends over time which were further exacerbated by the COVID-19 pandemic, particularly in those with certain co-occurring long-term mental health conditions. Compared to earlier time periods, the risk of deaths in Black Caribbean and Black African populations with SMI, increased during the pandemic. The reasons underlying observed disparities are multiple (e.g. stigma and diagnostic overshadowing [Shefer, Henderson, Howard, Murray, & Thornicroft, 2014], reduced access to care) and should be explored in future work (Impara et al., 2022). Our findings highlight a need to consider the additional vulnerabilities which impact the risk of death in SMI populations, which have been further magnified during the COVID-19 pandemic.

## Supporting information

Das-Munshi et al. supplementary material Das-Munshi et al. supplementary material

## Supplementary material

The supplementary material for this article can be found at https://doi.org/10.1017/S0033291724001843
