# Supplementary material for: Long term mortality trends in people with severe mental illnesses and how COVID-19, ethnicity and other chronic mental health comorbidities contributed: a retrospective cohort study
Source: Psychol Med. 2024 Oct 21;54(13):3678–88. doi: 10.1017/S0033291724001843 (PMC11536139; doi:10.1017/S0033291724001843)
Supplement: Das-Munshi et al. supplementary material [file S0033291724001843sup001.docx]

**Supplementary material**

**TITLE:** Long term mortality trends in people with severe mental illnesses (SMI) and how COVID-19, ethnicity and other long-term mental health conditions contributed: A retrospective cohort study

**Authors:** Jayati Das-Munshi, Ioannis Bakolis, Laia Bécares, Hannah Dasch, Jacqui Dyer, Matthew Hotopf, Rosie Hildersley, Josephine Ocloo, Robert Stewart, Ruth Stuart, Alex Dregan

**Supplementary tables:** 2

**Supplementary figures:** 5

**Supplementary material**

**Figure 1: STROBE flow diagram of cohort**


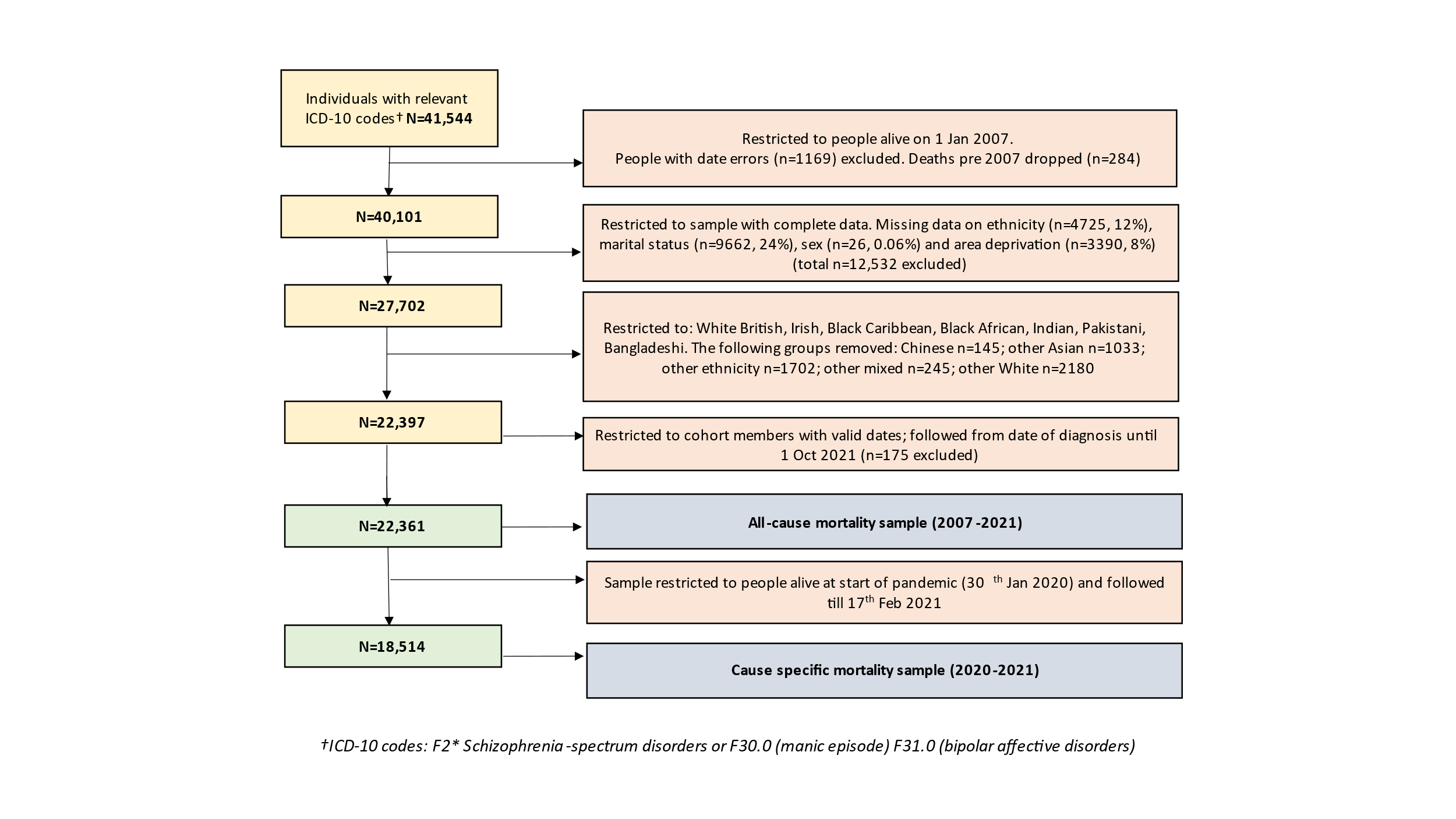


**Supplementary material**

**Figure 2: Sensitivity analyses: All-cause mortality by comorbidity**

*Supplementary analyses: Latest entry into the cohort (by date of SMI diagnosis) was restricted to 31^st^ December 2010.*

**Supplementary material**

**Figure 3: Sensitivity analyses: All-cause mortality by ethnicity**

*Supplementary analyses: Latest entry into the cohort (by date of SMI diagnosis) was restricted to 31^st^ December 2010.*

**Supplementary material**

**Table 1**

Table displaying association of SMI (total sample and with comorbidities) with risk of death. Time period modelled as a linear variable

|  | **All-cause mortality risk***  **RR (95% CI)** | **p-value** |
| --- | --- | --- |
| **Total sample** | 1.01 (1.00, 1.02) | 0.003 |
| **SMI comorbidity** |  |  |
| Depression | 1.03 (1.02, 1.05) | p<0.001 |
| Dementia | 1.12 (1.09, 1.14) | p<0.001 |
| Learning disability | 1.04 (1.00, 1.08) | 0.033 |
| SUD | 1.05 (1.03, 1.07) | p<0.001 |
| Anxiety | 1.10 (1.07, 1.12) | p<0.001 |

**Time period modelled as a linear/ quantitative indicator, from 2007 to 2021. (Time periods were: -2007; 2008-2009; 2010-2011;2012-2013;2014-2015;2016-2017;2018-2019;2020-2021). All estimates have been adjusted for age, marital status, sex, area deprivation, ethnicity and time since diagnosis. SUD: Substance and alcohol use disorders*

| **Assessment of time period (linear) (displayed in supplementary table 1) vs. time period (categorical) (displayed in Figure 1)**  Likelihood Ratio Tests (LRT) were used to assess the fit of models with time period modelled as a categorical variable (i.e. no assumption about how log rate changed with time) and time period as a quantitative variable (i.e. linear assumption of log rate change), with a null hypothesis that the effect of time period is linear. LRT p-values for these models were: total sample p=0.0036; depression p=0.038; dementia p=0.0022; learning disability p=0.010; substance and alcohol use disorders p=0.0093; anxiety p=0.014.  Each of the LRTs provided stronger support for ‘time period’ as a categorical variable over a linear variable. |
| --- |

**Supplementary material**

**Table 2: Association between comorbidities in severe mental illnesses and all-cause mortality, multiple imputation estimates**

| **Comorbidity** | **Year** | **IRR** | **(95%** | **CI)** |
| --- | --- | --- | --- | --- |
| Total sample | -2007 | 0.91 | (0.77, | 1.08) |
| Total sample | 2008-2009 | 1.14 | (1.01, | 1.28) |
| Total sample | 2010-2011 | 1.08 | (0.97, | 1.21) |
| Total sample | 2012-2013 | 1.02 | (0.92, | 1.14) |
| Total sample | 2014-2015 | 1.00 | REF |  |
| Total sample | 2016-2017 | 0.99 | (0.89, | 1.10) |
| Total sample | 2018-2019 | 1.02 | (0.92, | 1.13) |
| Total sample | 2020-2021 | 1.20 | (1.08, | 1.32) |
| Depression | -2007 | 0.72 | (0.54, | 0.96) |
| Depression | 2008-2009 | 0.92 | (0.77, | 1.11) |
| Depression | 2010-2011 | 0.96 | (0.81, | 1.14) |
| Depression | 2012-2013 | 0.95 | (0.81, | 1.12) |
| Depression | 2014-2015 | 1.00 | REF |  |
| Depression | 2016-2017 | 0.98 | (0.84, | 1.14) |
| Depression | 2018-2019 | 1.04 | (0.90, | 1.21) |
| Depression | 2020-2021 | 1.29 | (1.12, | 1.48) |
| Dementia | -2007 | 0.36 | (0.21, | 0.63) |
| Dementia | 2008-2009 | 0.47 | (0.33, | 0.66) |
| Dementia | 2010-2011 | 0.66 | (0.50, | 0.88) |
| Dementia | 2012-2013 | 0.89 | (0.69, | 1.15) |
| Dementia | 2014-2015 | 1.00 | REF |  |
| Dementia | 2016-2017 | 1.15 | (0.90, | 1.47) |
| Dementia | 2018-2019 | 1.21 | (0.95, | 1.55) |
| Dementia | 2020-2021 | 2.02 | (1.60, | 2.56) |
| Learning Disability | -2007 | 0.40 | (0.12, | 1.32) |
| Learning Disability | 2008-2009 | 0.94 | (0.53, | 1.66) |
| Learning Disability | 2010-2011 | 1.03 | (0.62, | 1.72) |
| Learning Disability | 2012-2013 | 0.56 | (0.31, | 1.01) |
| Learning Disability | 2014-2015 | 1.00 | REF |  |
| Learning Disability | 2016-2017 | 0.89 | (0.54, | 1.46) |
| Learning Disability | 2018-2019 | 0.80 | (0.49, | 1.33) |
| Learning Disability | 2020-2021 | 1.46 | (0.93, | 2.28) |
| SUD | -2007 | 0.79 | (0.48, | 1.29) |
| SUD | 2008-2009 | 0.67 | (0.47, | 0.96) |
| SUD | 2010-2011 | 0.94 | (0.70, | 1.27) |
| SUD | 2012-2013 | 1.05 | (0.80, | 1.39) |
| SUD | 2014-2015 | 1.00 | REF |  |
| SUD | 2016-2017 | 1.19 | (0.92, | 1.54) |
| SUD | 2018-2019 | 1.34 | (1.05, | 1.72) |
| SUD | 2020-2021 | 1.54 | (1.21, | 1.96) |
| Anxiety | -2007 | 0.50 | (0.25, | 0.99) |
| Anxiety | 2008-2009 | 0.44 | (0.28, | 0.71) |
| Anxiety | 2010-2011 | 0.62 | (0.43, | 0.91) |
| Anxiety | 2012-2013 | 0.66 | (0.47, | 0.94) |
| Anxiety | 2014-2015 | 1.00 | REF |  |
| Anxiety | 2016-2017 | 1.09 | (0.83, | 1.44) |
| Anxiety | 2018-2019 | 1.25 | (0.96, | 1.63) |
| Anxiety | 2020-2021 | 1.42 | (1.09, | 1.83) |

**Supplementary material**

**Figure 4: Sensitivity analyses: All-cause mortality by comorbidity, restricted to schizophrenia-spectrum diagnoses.**

*Supplementary analyses: Analyses have been restricted to people with schizophrenia-spectrum (ICD-10 F2*) diagnoses. Displayed adjusted rate ratios (RRs) are for the total sample and stratified by comorbidity, relative to the 2014-2015 reference period. Red estimates denote adjusted rate ratios for all-cause mortality from 30^th^ January 2020-2021 (first year of the COVID-19 pandemic). All estimates adjusted for age, marital status, gender, area deprivation and time since SMI diagnosis.*

**Supplementary material**

**Figure 5: Sensitivity analyses: All-cause mortality by ethnicity, restricted to schizophrenia-spectrum diagnoses.**

*Supplementary analyses: Analyses have been restricted to people with schizophrenia-spectrum (ICD-10 F2*) diagnoses. Displayed adjusted rate ratios (RRs) are for the total sample and stratified by ethnicity relative to the 2014-2015 reference period. Red estimates denote adjusted rate ratios for all-cause mortality from 30^th^ January 2020-2021 (first year of the COVID-19 pandemic). All estimates adjusted for age, marital status, gender, area deprivation and time since SMI diagnosis.*
